# Time budget, oxygen consumption and body mass responses to parasites in juvenile and adult wild rodents

**DOI:** 10.1186/s13071-016-1407-7

**Published:** 2016-03-01

**Authors:** Mario Garrido, Valeria Hochman Adler, Meital Pnini, Zvika Abramsky, Boris R. Krasnov, Roee Gutman, Noga Kronfeld-Schor, Hadas Hawlena

**Affiliations:** Mitrani Department of Desert Ecology, Swiss Institute for Dryland Environmental and Energy Research, Jacob Blaustein Institutes for Desert Research, Ben-Gurion University of the Negev, Midreshet Ben-Gurion, Israel; Department of Life Sciences, Ben-Gurion University of the Negev, Beer-Sheva, Israel; Department of Zoology, Tel Aviv University, Tel Aviv, Israel; Departments of Animal Science and Nutritional Sciences, Faculty of Sciences and Technology, Tel-Hai College, Upper Galilee, Israel; Unit of Integrative Physiology (LIP), Laboratory of Human Health and Nutrition Sciences, MIGAL – Galilee Research institute, Kiryat Shmona, Israel

**Keywords:** Body mass, Compensatory responses, Ectoparasites, Energy budget, Fleas, Host age, Metabolic rate, Rodents, Time budget

## Abstract

**Background:**

The study of changes in a host’s energy allocation in response to parasites is crucial for understanding parasite impact on both individual- and population-level processes. Experimental studies have explored such responses mainly in a single subsample of hosts per study, primarily adult males, and have only assessed either the overall energy acquisition or expenditure, rather than their different components simultaneously, or the behavioral responses. Accordingly, two fundamental questions arise: why have multiple host strategies evolved to cope with increased energy expenditure? and, which factors determine this variation (e.g. host species, identity, age)? This study provides an important step towards addressing both questions by experimentally disentangling the short-term physiological and behavioral responses of juvenile and non-reproductive adult rodents to natural levels of flea infestation. These two cohorts represent extreme cases of the energy demand continuum, as the former, in contrast to the latter, is involved in growth - a highly energy-demanding process - and may not be able to operate far below its upper limit of energy expenditure, and thus should reduce its energy expenses upon the occurrence of extra demands (e.g. due to parasitic pressure). Accordingly, we hypothesized that the response to fleas is age-dependent and varies according to the age-specific energy requirements and constraints.

**Methods:**

We monitored the behavior and physiology of juvenile and non-reproductive adult rodents before and after experimental flea infestation. First, we used a model selection approach to search for the factors that best explained the variability in the time budget, oxygen consumption, and body mass change in response to fleas. Then, using a path analysis approach, we quantified the different pathways connecting the important associations revealed at stage 1.

**Results:**

Compared to their flea-free counterparts, flea-infested adults groomed longer and had a higher oxygen consumption rate, but did not lose body mass. Infested juveniles also groomed longer but grew slower and had a similar rate of oxygen consumption.

**Conclusions:**

Results suggest that both juvenile and adult rodents suffer from natural flea infestation levels. However, the comparison between the responses of juveniles and adults to experimental infestation, also suggests that juveniles may reallocate their energy expenditure from growth to maintenance, while non-reproductive adults increase their energy acquisition. Such age-dependent responses suggest that juveniles may be constrained by their higher need to rest for full functioning or by an upper limit in energy expenditure. Taken together, our study provides experimental evidence that hosts can compensate for the costs incurred by parasitism through physiological and behavioral plasticity, depending on their age, which probably determines their requirements and constraints. These compensatory responses may have important implications for the population dynamics of hosts and their parasites.

## Background

The reproductive success of an individual and its interaction with other individuals is largely determined by its use of energy (e.g., [1–3]). Thus, the study of changes in a host’s energy allocation in response to parasites is crucial for understanding parasite impact on both individual- and population-level processes [1, 2].

**Fig. 1:**
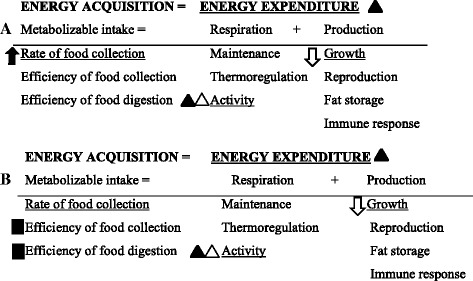
Predicted and observed changes in the energy budget of flea-infested rodents. Predicted (**a**) and observed (**b**) changes in the energy budget of juvenile and adult rodents under natural infestation levels due to energy consumption of fleas and flea-induced behavioral changes (filled and empty triangles for adults and juveniles, respectively) and compensatory response by juvenile (empty arrows) and adult (filled arrows) hosts (reproduced from Munger & Karasov, [1]). All relevant components are considered, while only underlined components were directly tested during the study. Filled squares indicate the compensatory responses by adults supported, but not proven, by the results

Energy acquisition is a function of both the availability and quality of food consumed and of the rate and efficiency of food collection and digestion (Fig. 1). This acquired energy is used for maintenance, thermoregulation, and activity, and for tissue production and biological processes, such as growth, reproduction, fat storage, and immunity (Fig. 1). Parasites derive their food from their host, thereby directly increasing the host’s energy expenditure [4–7]. In addition, parasites may induce changes in host behavior and immune response (including inflammatory response), which further indirectly increases the host’s energy expenditure [8–13]. To keep energy homeostasis, the total energy acquired must equal the total energy expended. An increase in the latter is expected to result in insufficient energy allocated to satisfy all the energy requirements, including maintenance (Fig. 1). However, since an energy shortage for maintenance often results in irreversible damage, the host must compensate for the negative impact of parasites by changing its available energy allocation.

Accordingly, some species offset any increased energy requirements, including the negative effect of parasites, by increasing their metabolizable intake (Fig. 1; [1, 12, 14, 15]). For example, various rodent and bird species increase their food consumption and provisioning for their offspring when experimentally infected with parasites [16–20]. In these cases, often, the energy expenditure of infested individuals is higher than that of their parasite-free counterparts, as reflected in the differences in oxygen consumption rates between experimental and control groups. However, infested individuals are able to maintain a stable body mass, body temperatures and/or reproductive efforts (e.g. [21–24]).

However, organisms that are prone to remarkably high energy-demanding processes, such as reproductive individuals or those exposed to stressful situations (e.g., high predation risk), cannot afford to further increase their metabolizable intake since they are already operating close to the upper limit of their ability to utilize, ingest, and store energy for metabolism [24–28]. These organisms may thus use an alternative strategy to compensate for the parasitic effect by reducing their energy expenditure (Fig. 1; [14, 15]). This could be done, for example, by reducing the energy allocated to growth [24, 29], fat storage [30], reproduction [31] and thermoregulation [1, 32–34]. As a result, under parasitic pressure, hosts using the latter strategy have an energy expenditure rate similar to their parasite-free conspecifics [1, 32, 35, 36].

At present, experimental studies have explored strategies for energy compensation in a single host type, mainly non-reproductive adult male hosts (e.g. [32, 37, 38], but see, for example, [29, 33, 35]). Such subsamples of a host population used in the laboratory experiments may represent simply a particular case of hosts, whereas other host types (e.g. adult females, juveniles, reproductive hosts) may suffer from and respond to parasites differently [39–42]. The focus on a single host type has also restricted our ability to explore how host energy requirements and constraints may influence their response to parasitism. In addition, in most studies, either the overall energy acquisition or expenditure, rather than their different components, was quantified (e.g. [8, 35, 39]). This approach makes it difficult to distinguish between direct parasite effects and host responses, and it limits our mechanistic understanding of host strategies to cope with parasite effects. Accordingly, two fundamental questions arise: (i) why have multiple host strategies evolved to cope with the increased energy expenditure associated with parasitism? and (ii) which factors determine the exact host strategy (e.g. host species identity, age, etc.)?

This study provides an important step toward addressing these two questions by disentangling the responses of juvenile and non-reproductive adult rodents (*Meriones crassus*) to flea (*Xenopsylla conformis*) infestation and separating them into behavioral and physiological components. Juveniles and non-reproductive adults may represent two extreme cases of the energy demand continuum. Adult non-reproductive rodents are likely to operate far below their upper limit of energy expenditure, whereas juveniles must pay the costs of growth, a highly energy-demanding process; therefore, juveniles are expected to live near their upper energy limit and to maximize their energy utilization from the environment as well as maximizing their internal system functioning [24, 26, 27, 43–46]. Thus, juveniles cannot further increase their energy acquisition when a new demand arises and might instead reallocate their energy expenditure. Therefore, we hypothesized that flea infestation would increase the energy expenditure in juvenile and adult hosts, both directly and indirectly, *via* induced grooming [8, 47, 48]. We also hypothesized that the response to parasites would be different in the two age groups due to differential age-dependent energy requirements and constraints [39, 43, 49]. In particular, we expected adults to compensate for flea effects by increasing their metabolizable intake and juveniles to reduce their energy expenditure (Fig. 1).

We simultaneously monitored the behavior, oxygen consumption, and body mass of juvenile and adult rodents before and after flea infestation, aiming to reveal whether the two groups would have different changes in time budget, energy acquisition, and energy expenditure in response to fleas. Through quantifying short-term host responses to 48 h of flea infestation, we emulated a common phenomenon in the field, in which rodents occupy flea-infested burrows or encounter a substantial load of host-questing fleas and need to respond to this challenge immediately. This short-term flea manipulation also allowed us to avoid the potential induction of an adaptive immune response, and thus, to isolate the physiological and behavioral responses, measured in energy currency. By comparing the observed patterns to a null model, and by combining model selection and path analysis approaches, we collected evidence supporting our hypothesis that while flea infestation causes direct and indirect energy-demanding changes in the physiology and behavior of both juvenile and adult rodents, the compensation strategy is age-dependent.

## Methods

### Study system

#### Rodents

*Meriones crassus* Sundevall is a common rodent species in southern Israel. We used rodents from our laboratory colonies. Progenitors of the colony were captured at the Ramon erosion cirque, Israel (30°35’N, 34°45’E) in 1996. We used 24 non-reproductive adult (140.95 ± 2.91 g) and 18 juvenile (35.47 ± 1.47 g) rodents. All rodents were immunologically naïve males to control for possible differences due to previous exposure to fleas [50] and sexual [51] biases. Juvenile rodents were separated from their mothers 30 d postpartum, and after 3 d in a cage with other siblings, they were placed individually in the experimental cages. At this age, the juveniles were already weaned and since they were kept separately, the two age-groups faced similar conditions (e.g. no social interactions, requirements to search for seeds in the sandy substrate, and no exposure to predator risk, excluding our daily visits), other than the physiological need of juveniles to grow. From each litter, a sibling couple was randomly selected, and then, the two males were randomly assigned to the two experimental groups. This design reduced the genetic variability between the two experimental groups, thus increasing the sensitivity of our assays to detecting the physiological and behavioral responses to flea infestation. Adult hosts, at least six months old, were each housed individually both prior to and during the experiment to ensure that they were not reproductively active. We did not have pairs of male siblings in adult rodents, which prevented us from controlling for genetic variability in adults.

Prior to the experiments, all rodents were allowed seven days of acclimation in the experimental cages within the experimental room and an additional day during which the respiratory system was operated. During the experiments, the animals were housed individually in glass cages (21 × 31 × 13 cm for adults and 17.5 × 28 × 13 cm for juveniles) with covered sidewalls that enabled clear observation of their behavior but completely prevented the animals from seeing each other. The floor in each cage was covered with 1 cm of sand, and each cage contained *ad libitum* millet seeds and 4 g of alfalfa as a water source. Animals were maintained at 28 ± 1 °C with a photoperiod of 12:12 h (light:dark). In the dark, the room was lit with a dim red light. This experimental setting emulated the field conditions for both the rodents and the fleas as the rodents gained their energy, nutrients, and water from seeds and leaves, and they searched for seeds in the sand, where the fleas could attack a host during a host-flea encounter.

#### Fleas

*M. crassus* is naturally parasitized by several flea species; *Xenopsylla conformis* is a characteristic parasite of this rodent [52]. Fleas were obtained from laboratory colonies, started in 1998–2001, from field-collected specimens on *M. crassus* using rearing procedures described elsewhere [41]. Colonies of fleas were maintained at 25 °C and 75 % relative humidity, with a photoperiod of 12:12 h (light:dark).

### Experimental design

Adult and juvenile rodents were randomly subjected to two treatments; 50 % of the animals from each group were flea-infested (treatment), and 50 % were left flea-free (control). Each experiment trial lasted for four consecutive days with a similar experimental procedure including four days of oxygen consumption measurements, two of which also included behavioral observations. However, during the first two days of each trial, no fleas were introduced to any of the animals, whereas during the third and fourth days, rodents from the treatment group were infested with fleas. This experimental design allowed us to distinguish between the effects of acclimatization (comparison of the control group on the first two and last two days) and fleas (comparison of the temporal changes between the treatment and control groups) on the oxygen consumption and behavior of the rodents.

#### Flea manipulation

We placed fleas on the treatment rodents at the onset of the third day of each trial, 1 h prior to the first observation. Initial flea numbers were standardized according to the surface area of the rodents to allow equal densities on all rodents (calculated as the number of fleas divided by the rodent body mass to the power of 0.67; [53]). Then, at the onset of the fourth day, we added half of the initial flea numbers to each treated rodent to compensate for flea mortality, which is estimated to be, under lab conditions, 50 % (Hawlena et al. unpublished data). Thus, the estimated daily number of live fleas per rodent was similar across all individuals and experimental days. In nature, *X. conformis* prevalence is mostly 100 %, and the mean infestation on an *M. crassus* individual ranges from 3 to 21, depending on the season and habitat, with some individuals harboring more than 50 fleas [52, 54, 55]. Considering that these numbers were calculated jointly for juveniles and adults, and that in the rodent burrows, there can be 30–100 fleas more (Shenbrot G, personal communication), the flea numbers that we added to the host cages (from 41–66 and 113–135 for juvenile and adult hosts, respectively) represented the higher end of the infestation level range that *M. crassus* may naturally face.

#### Behavioral observations

The behavior of each individual rodent was monitored before and after the treatment was applied. Each time, rodents were filmed over 24 h, for 20 min per hour, using infrared video cameras. Following Hawlena et al. [47], we distinguished between four major activities: (1) feeding (eating seeds and alfalfa), (2) grooming, including both grooming and scratching (moving the extremities over the body and mouthing the body and extremities), (3) “other activities” (any activity in the cage that was not targeted to feeding or grooming, including movement between different locations, sand removal, and vigilance), and (4) resting (corresponding to both standing still and sleeping, which were not distinguishable). The reported data was calculated as the mean total time spent in each activity per 12 h (day and night). In addition, to assess the rate of food collection (Fig. 1), we calculated the duration and frequency of the feeding bouts. These were considered to be terminated when either a change in activity was detected, or at the end of the observation period. Such truncation of the bouts at the end of the observation period may have caused an underestimation of the mean feeding duration, but this was assumed to affect all rodents in a similar way.

#### Oxygen consumption and body mass

Hosts were weighed to 0.01 g before being placed in the respiratory system and at the end of each trial. The rate of change in the body mass of each individual was calculated as body mass after trial minus body mass before trial, and divided by the number of days of the experiment. Oxygen consumption rates (ml ⋅ g^− 1^ ⋅ min^− 1^) were measured in an open respiratory system, following Depocas & Hart [56] and were used as indirect indicators for overall energy expenditure. *V*O_2_ was calculated using Withers [57], following eq. 1:

1 $$ V{O}_2={V}_2\times \left({F}_1{O}_2-{F}_2{O}_2\right)/\left(1-{F}_1{O}_2\right) $$

where *V*_2_ is the rate of airflow in the chamber (ml ⋅ min _STPD_^− 1^), *F*_1_O_2_ is the fractional concentration of O_2_ entering the cage, and *F*_2_O_2_ is the fractional concentration of O_2_ in the outflowing air. STPD is standard temperature (0 °C), pressure (760 mm Hg), and dry air (see [58] for more details). Airflow through the cages was 400–430 ml · min^−1^ for adults and 300–330 ml · min^−1^ for juveniles. Average oxygen consumption rates were calculated for a period of 12 h.

### Ethical approval

The experimental protocol met the requirements of the 1994 Law for the Prevention of Cruelty to Animals (Experiments on Animals) of the State of Israel and was approved by the Ben-Gurion University Committee for the Ethical Care and Use of Animals in Experiments.

### Null model for a behavior-mediated effect of fleas on oxygen consumption

To distinguish between the direct and behavior-mediated effects of fleas, we compared the observed oxygen consumption with the expected consumption, based solely on behavioral changes. The expected diurnal and nocturnal consumption rates (Exp O_2_), before and after the treatment was applied, were calculated for each individual, following eq. 2:

2 $$ \mathrm{E}\mathrm{x}\mathrm{p}\ {\mathrm{O}}_2={\displaystyle {\sum}_i^4{T}_i\times {C}_i} $$

where “T” is the total time devoted for each of the *i*th activities (in minutes) and measured “C” is the energetic cost for each of the *i*th activities (in ml ⋅ g^− 1^ ⋅ min^− 1^). The diurnal and nocturnal energetic costs for the *i*th activity (*C*_*i*_) were assessed for each individual before the infestation treatment was applied, following eq. 3:

3 $$ {C}_i={O_2}_i-{O}_{2_s} $$

where “${O}_{2_i}$” is the oxygen consumed per gram of body mass when performing the *i*th activity and ${O}_{2_s}$ is the oxygen consumed per gram of body mass when sleeping. For the energetic cost calculations, we first matched an observed behavior with the associated intrinsic oxygen consumption by subtracting the 11 min and 7 min time lags between the oxygen and behavioral records of juveniles and adults, respectively (Garrido et al. unpublished data).

### Statistical analyses

Analyses were conducted in two stages: first, we searched for the most important factors that best explained the variability in the (i) O_2_ consumption, (ii) behavioral budget, and (iii) body mass change of rodents (Table 1); then we quantified the causal pathways of flea effects, using a path analysis approach [59]. For both stages, we compared models using model probabilities (*w*_i_, where i corresponds to a specific model) based on an Akaike information criterion corrected for a small sample size (AICc), which gives a measure of the plausibility, on a 0 to 1 scale, that a particular model is indeed the best model [60].

**Table 1:** Comparison of models from stage 1

Effect tested	Dependent variables					
	Oxygen consumption	Body mass change	Resting	Feeding	Grooming	Other activities
1. Null model	0	0	0	0	0	0
2. Treatment × Experimental time(ET)	0	0	0	0	0	0
3. Treatment × ET + Activity	0	NA	NA	NA	NA	NA
4. Treatment × ET × Activity	0	NA	NA	NA	NA	NA
5. Activity	0	NA	NA	NA	NA	NA
6. Age	NA	0	NA	NA	NA	NA
7. Treatment × ET × Age	0	**100 %**	0	0	0	0
8. Treatment × ET × Time of the day (TD)	0	NA	0	0	0	0
9. Treatment × ET × TD × Age	0	NA	**100 %**	**100 %**	**100 %**	**100 %**
10. Treatment × ET + Age	NA	0	NA	NA	NA	NA
11. Treatment × ET × TD × Age + Activity	**100 %**	NA	NA	NA	NA	NA

Model selection results of comparison between models describing the variability in the oxygen consumption (ml ⋅ g^− 1^ ⋅ min^− 1^), body mass change (%), and the time (min) devoted to the four major activities of juvenile and adults rodents. Values are weights (*w*_i_) in percentages of Akaike information criterion corrected for sample size—the relative likelihood of the current model, given the data and the set of models. The set of models includes the null model (all relevant effects), the pure treatment effect model (#2; the interaction between flea-infested and flea-free rodents and the experimental time (first two versus last two days of experiment)), and additive or multiplicative effects of the flea treatment, with age (juveniles versus adults), activity (total time being active), and the time of the day (light versus dark hours). In all models that include interaction terms, the lower order interactions were included although not shown. The best models supported by the data (w_i_ > 20 %) are marked in bold and were used for stage 2

*NA* Not applicable

At stage 1, each model in the model set was a linear mixed-effect model (LMM), with a normal distribution of each of the three dependent variables (i-iii) and rodent identity as the random factor in all cases (to account for repeated measurements). The fixed explanatory variables in each model are presented in Table 1. At stage 2, each model described different pathways connecting the important associations revealed at stage 1. Path analysis is a powerful approach that evaluates alternative causal hypotheses regarding the interactions among variables. The causal links that this analysis reveals are often supported by an experimental approach [59]. The model selection approach complements the path analysis by evaluating the likelihood of the causal hypotheses, which reflect different predictions about the directions and strength of interactions, given the data and the set of models (see [61] as an example). To this end, in addition to the insights gained from traditional regression-based approaches about the strength and significance of pairwise interactions, the combined path analysis-model selection approach can incorporate multiple interrelated dependent variables (e.g. oxygen consumption and activity time), can predict the direction and causality of the interactions, and can distinguish between direct and indirect effects (e.g. flea effects on oxygen consumption mediated by behavioral changes).

The path analysis was conducted using the Mplus software [62]; Los Angeles, CA, USA), whereas all other analyses were conducted with the program R, version 3.1.3 (packages nlme, MuMIn, and lme4; [63–66]).

## Results

### Oxygen consumption and body mass

**Fig. 2:**
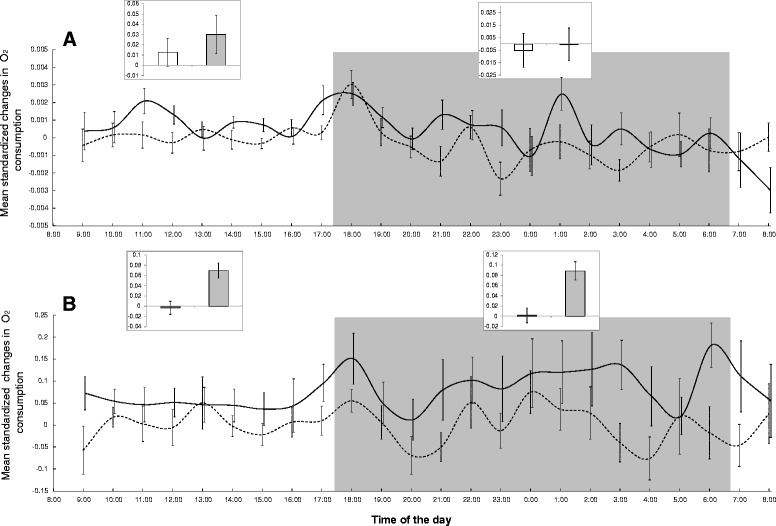
Oxygen consumption changes of flea-infested and flea-free juvenile and adult rodent hosts. Mean (±SE) standardized changes between post- and pre-experimental days in oxygen consumption of flea-infested (solid line) and flea-free (dashed line) *Meriones crassus* juveniles (**a**, *N* = 18) and adults (**b**, *N* = 24) per hour (main figures) and at 12-hour scales (four inserts). Standardized changes are measured as the proportion of differences in oxygen consumption rate (ml O_2_ ⋅ g^− 1^ ⋅ min^− 1^ per g of rodent) between post- and pre-experimental days (each is a mean of 48 h) to the consumption rate in the two pre-experimental days. The grey background corresponds to the dark hours

The variability in rodent oxygen consumption was best explained by the four-way interaction between treatment (flea-infested versus flea-free rodents), experimental period (first two versus last two days of experiment), the time of the day (light versus dark hours), and age (juveniles versus adults), and the additive effect of activity time (Table 1). In particular, the oxygen consumption of adults, but not of juveniles, significantly increased by 7.2 ± 2.8 % due to flea addition, and this effect was most pronounced during the night (6.5 ± 3.2 % and 8.0 ± 4.8 % increase in day and night, respectively; Fig. 2). The inclusion of activity time in the best model suggests that at least part of the flea effect is mediated by changes in the rodent activity (section 3.3).

**Fig. 3:**
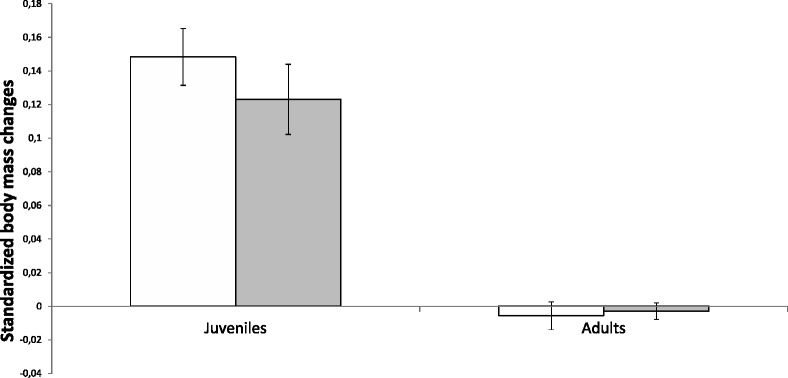
Body mass changes of flea-infested and flea-free juvenile and adult rodent hosts. Mean (±SE) standardized body mass changes between post- and pre-experimental days of flea-free (white) and flea-infested (grey) juvenile and adult *Meriones crassus*. Standardized changes are measured as the proportion of body mass (g) change between post- and pre-experimental days to the initial body mass

The variability in rodent body mass was best explained by the three-way interaction between treatment, experimental time, and age (Table 1). In particular, flea-infested juveniles grew slower than their flea-free counterparts did in the two post-as compared with the two pre-experimental days (*w*_i_ = 100 % for the two-way interaction of only the juvenile data; Fig. 3). In contrast, the body mass change of flea-infested and flea-free adult rodents was similar whether they were infested or not (*w*_i_ =0 % for the two-way interaction of only the adult data; Fig. 3).

### Behavioral time budget

**Fig. 4:**
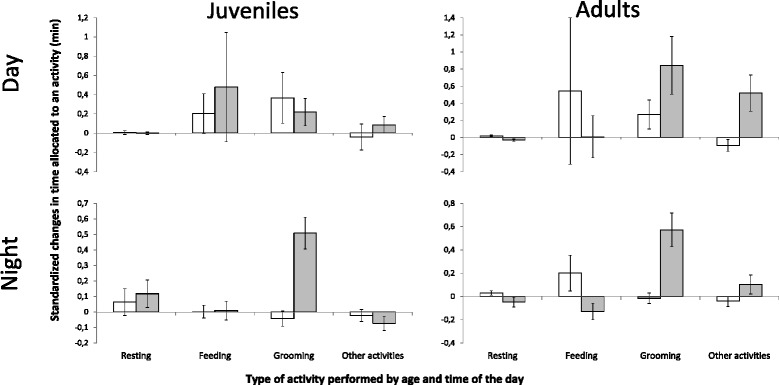
Changes in the time budgets of flea-infested and flea-free juvenile and adult rodent hosts. Mean (±SE) standardized changes between post- and pre-experimental days in time allocated to resting, feeding, grooming, and performing other activities by flea-free (white) and flea-infested (grey) juvenile and adult *Meriones crassus*. Standardized changes are measured as the proportion of differences in time allocated to each behavior (min) between post- and pre-experimental days to the time allocated in the pre-experimental day

The variability in the total time allocated to each of the four activities was best explained by the four-way interaction between treatment, experimental time, the time of the day, and age (Table 1). While flea infestation affected the time allocation amongst the four activities in both juveniles and adults, the change in behavior was more pronounced in adults (Fig. 4). In particular, flea-infested adults increased their time allocation to grooming and performing other activities at the expense of feeding and resting (Fig. 4). Moreover, the change in time spent grooming, feeding, and resting was more pronounced in the dark than in the light hours (Fig. 4). The effect of fleas on the time budget of rodents was reflected mainly in the increased time allocated to grooming (Fig. 4). However, in contrast to our predictions, flea-infested adults spent less time feeding during nighttime, and the mean duration and frequency of feeding bouts remained constant in all cases (data is shown only for total feeding time; Fig. 4).

### Direct versus behavior-mediated effects of fleas on oxygen consumption

**Fig. 5:**
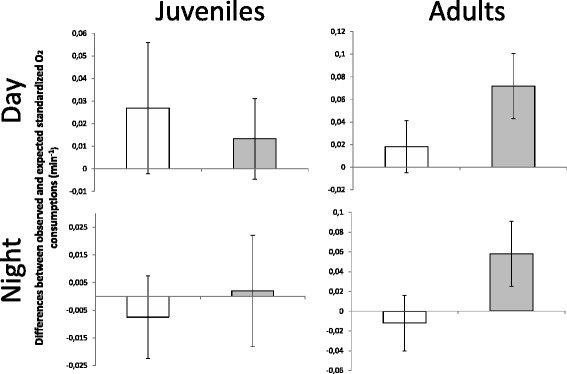
Null model for a behavior-mediated effect of fleas on oxygen consumption. Mean (±SE) differences between observed and expected standardized changes in O_2_ consumption rates (ml ⋅ g^− 1^ ⋅ min^− 1^) between post- and pre-experimental days by flea-free (white) and flea-infested (grey) juvenile and adult *Meriones crassus*. Standardized changes are measured as the proportion of differences in consumption between post- and pre-experimental days to the consumption in the two pre-experimental days. Expected changes are based solely on behavioral changes due to flea effects (see section 2.3)

The variability in oxygen consumption change between the post-and pre-experimental days was best explained by the two-way interaction between treatment and data source (observed or expected), and by the three-way interaction between the treatment, data source and age (Table 2). As expected, all flea-free rodents showed similar observed and expected oxygen consumption changes, and flea-infested juveniles demonstrated similar patterns (Fig. 5). However, flea-infested adults showed a higher increase in oxygen consumption due to flea addition than the expected effect caused by behavioral changes alone (Fig. 5).

**Table 2:** Comparison of models explaining the oxygen consumption variability between the observed and the expected solely by behavioral changes

Effect tested	*w*_i_
1. Null model	1 %
2. Treatment	4 %
3. Data source (DS)	9 %
4. Treatment × DS	**45 %**
5. DS × Age	4 %
6. DS × Time of the day (TD)	3 %
7. Treatment × DS × Age	**32 %**
8. Treatment × DS × TD	2 %
9. Treatment × DS × TD × Age	0

Model selection results of comparison between nine models describing the variability in the observed and expected oxygen consumption (ml ⋅ g^− 1^ ⋅ min^− 1^) change between post-and pre-experimental days for juvenile and adult *Meriones crassus*. Values are weights (*w*_i_) in percentages of Akaike information criterion corrected for sample size—the relative likelihood of the current model, given the data and the set of models. The set of models includes the null model (#1), the pure flea effect model (#2), the data source (DS; observed or expected, #3) and the additive or multiplicative effects with age (juveniles versus adults), treatment (control versus flea-infested rodents), and the time of the day (light versus dark hours). In all models that include interaction terms, the lower order interactions were included although not shown. The best models supported by the data (wi > 20 %) are marked in bold and were used for stage 2

### Causal pathways in flea effects on hosts

**Fig. 6:**
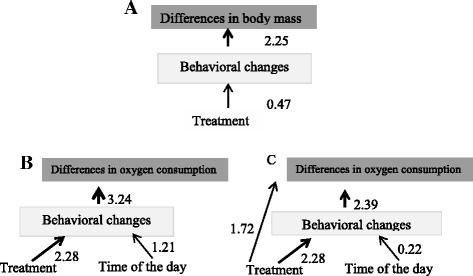
The best path analysis models explaining the different pathways of flea effects on hosts. The three best path analysis models describing the most important direct and/or indirect pathways of flea effects on the physiology and behavior of juvenile (**a**) and adult (**b** & **c**) *Meriones crassus*. Arrows represent direct and indirect influences. Numbers on the arrows are standardized path coefficients, representing the relative strength of the given effect (β/SE), which is also reflected by the arrow width

Based on the model selection results for flea effects on juvenile rodents, we compared three alternative models to explain the variability in body mass change. The first model included the direct effects of treatment on both body mass and behavioral changes. The second model included only an indirect effect of treatment, mediated by behavioral changes on body mass change (Fig. 6a). Finally, the saturated model included a mix of both direct and behavior-mediated effects. The variability in juvenile body mass was best explained by the indirect model (*w*_*i*_ = 70 %).

For adult rodents, we compared three alternative models to explain the variability in oxygen consumption. The first model included the direct effects of (i) treatment and of (ii) behavioral changes due to changes in the time of the day on oxygen consumption. The second model included only indirect effects, mediated by behavioral changes, and the effect of ii on oxygen consumption (Fig. 6b). Finally, the saturated model included a mix of both direct and behavior-mediated effects (Fig. 6c). The variability in adult oxygen consumption was best explained by the indirect and the mixed models, providing together good support of the data (∑*w*_*i*_ = 90 %). In contrast, the direct-effect model only provided weak support of the adult data (*w*_*i*_ = 10 %).

## Discussion

Our results suggest that juvenile and non-reproductive adult rodents perceive pressure incurred by fleas differently. A separation of juvenile and adult responses into their behavioral and physiological components further suggests that an age-dependent response to parasites is exhibited, probably due to the age dependency of ecological energy requirements and constraints. We discuss below our results in light of the two fundamental questions of which factors determine the exact host strategy, and why multiple strategies have evolved in response to the increased energy expenditure associated with parasitism.

### The host age influences its response to parasites

Host age plays an important role in determining host-parasite interactions as it affects the abundance and distribution of parasites among host individuals, the susceptibility and pathological levels of the individual hosts and the infestation success of parasites (e.g. [39, 41, 67–75]). Together with evidence on differential age-related resistance responses (e.g. [47, 76–78]), our study suggests that the host’s compensatory strategies for parasite effects are also age-dependent. In particular, while the infested adults increased their time spent grooming, had a higher oxygen consumption rate and did not lose body mass compared to flea-free counterparts, the infested juveniles, who also increased the time spent grooming, had a similar oxygen consumption rate to their flea-free counterparts. These results, together with the observed lower growth rate of the infested juveniles, support our hypothesis that juveniles reallocate their energy expenditure from growth to maintenance, while non-reproductive adults increase their energy acquisition, and thus do not lose body mass.

Nevertheless, the results only partly support our mechanistic prediction since evidence for increased energy acquisition by adults is only indirect, based on their increased oxygen consumption and stable body mass (Figs. 2 and 3), while infested adults did not show any increase in the frequency, duration, or total time spent feeding (Fig. 4). Alternative mechanisms for adults to acquire additional energy could include a diet shift toward the energy-richer food item (millet seeds at the expense of alfalfa) or an increase in their digestion efficiency, a similar mechanism to the one used by lactating females and non-reproductive adult *Peromyscus* rodents in cold winters [79]. However, our efforts to emulate the field conditions by using sand as a substrate and by providing the rodents with fresh leaves and seeds mixed with sand prevented us from quantifying the dry biomass consumed and ingested by the hosts to test the above mechanisms.

This evidence for age-dependent compensatory host responses emphasizes the importance of using multiple host types (e.g. sexes, ages, reproductive statuses) and multiple dependent variables (e.g. oxygen consumption, time allocated to various activities, and body mass) when experimentally testing parasite effect. For example, if we had assessed the effect of fleas solely on the oxygen consumption of juveniles or solely on the body mass change of adults, we would have concluded that natural flea infestation levels do not have a negative impact on rodents (e.g. [39]). Similarly, if we had solely focused on flea-related changes in time spent feeding (Fig. 4), we would have missed the changes in the energy acquisition of infested adults.

Other species-specific individual and population characteristic factors (e.g. the generation time of the host, its reproductive status, or predation risk) may also affect the strategies of energy compensation for parasite effects [39, 80–82], and thus should be considered in future studies. For example, the next step toward answering the two fundamental questions of why multiple host strategies have evolved to cope with increased energy expenditure, and which factors determine this variation would be to explore the causes for the age-dependent differences. Are they solely due to the higher energy demands of juveniles? If they are, should we expect that pregnant or lactating females or reproductive males would also show different compensatory strategies compared to non-reproductive female and male adults? Moreover, what will happen if we expose adults to stressful situations; will they change their compensatory strategy?

The exact mechanism underlying the intraspecific differences in response to stressful conditions, such as parasitism, remains unclear, but it may be hormone-regulated (e.g. [83–90]).

### Constraints associated with age-dependent compensatory responses

We collected evidence suggesting that fleas are energetically costly to both juvenile and non-reproductive adult rodents, partly because they induce changes in defense behaviors. If so, then why did juveniles not increase their energy acquisition to compensate for the flea effect as did adults? One possible explanation is that juveniles are constrained in their feeding rate and time since they are less efficient foragers, and should be more vigilant than adults [91–95]. However, this possibility is not relevant to our study because we offered food *ad libitum* to all rodents and no predators were present. Juveniles might also be constrained by their higher need to rest for full functioning, compared to adults [96, 97]. The failure to detect any reduction in resting time as a cost of grooming in infested juveniles, in contrast to infested adults, supports this explanation.

In addition, as mentioned above, non-reproductive adult rodents, similarly to most other organisms, are likely to operate far below their upper limit of energy expenditure, and thus are able to increase their expenditure under harsh conditions by elevating their energy acquisition [46, 79, 98–104]. The energy expenditure of juveniles, in contrast to adults, might already be near its upper limit. The approximately three times greater metabolic rates per mass unit of juveniles we recorded before flea addition supports our working assumption that juveniles have higher energy requirements for maintenance [49] and require additional energy for somatic growth and maturation [39]. Like other organisms undergoing highly energy-demanding processes [105–107], growing juveniles may have already reached the upper limit of their central processing organs related to feeding, digestion, and assimilation, or of their ability to utilize, ingest, and store energy for metabolism [2, 24, 44, 45, 79, 108, 109]. As a result, juveniles may not be able to further increase their energy expenditure in response to parasites, and must instead reallocate their energy expenditure. Our results suggest that they relocate their energy from growth to maintenance (Figs. 1 and 3).

Similar constraints are likely to cause interspecific or seasonal variability in host responses as well. For example, adult *Gerbillus dasyurus* rodents, similarly to juvenile *M. crassus*, do not increase their food intake but lose body mass under flea infestation, and infested *Gerbillus nanus* increase their oxygen consumption in spring and summer but not in winter [37, 110].

### Possible implications of the energy-related effects of parasites

It is commonly thought that co-evolved parasites, especially external ones, only generate a small, mainly indirect, energetic cost for their hosts [37, 111]. However, *via* a short-term laboratory experiment, we collected evidence showing that co-evolved fleas under natural levels of infestation (i) significantly increase the energy expenditure of their hosts, (ii) produce both direct and behavior-mediated costs, and (iii) induce different host responses, possibly according to age-dependent requirements and constraints. These three effects may have long-term implications for the ecology and evolution of the hosts. This is because *M. crassus*, like other rodents in the Negev Desert, has only two reproductive seasons; therefore, for most of their lives, adult rodents are not reproductive and have lower energy demands than juveniles [112]. First, an elevated energy expenditure may lead to great fitness costs, and the increased energy acquisition may come at the expense of other activities such as vigilance and mating [110, 113] thus decreasing the survival and reproductive success of adults [31, 114–116]. Second, the decrease in the juvenile growth rate due to flea addition may reduce their size as reproductive adults or may be linked to energy-costly compensatory growth [43, 117, 118]. However, to understand the exact long-term consequences of these energy-related effects, long-term manipulations should complement our short-term approach. This is because under longer infestation periods, adult hosts may mount an adaptive immune response (but see [67]) and/or increase their grooming efficiency, whereas fleas might limit the extent of host exploitation.

## Conclusion

In the present laboratory experiment, we simultaneously assessed both the physiological and behavioral short-term responses of juvenile and adult rodents to natural levels of flea infestation. We revealed that hosts could compensate for the costs caused by parasitism through physiological and behavioral plasticity, depending on their age, probably due to differences in their requirements and constraints. These compensatory responses may have important implications for the population dynamics of hosts and their parasites. Our study emphasizes the importance of using multiple host types (e.g. sexes, ages, reproductive statuses) and multiple dependent variables (e.g. oxygen consumption, time allocated to various activities, and body mass) when experimentally testing parasite effect.
